# TCF3 downregulation alleviates renal fibrosis via PI3K/Akt/mTOR pathway inhibition and autophagy restoration in diabetic nephropathy

**DOI:** 10.3389/fmed.2025.1547410

**Published:** 2025-12-01

**Authors:** Chen-xiao Liu, Yan-qiu Jiang, Ai-jie Huang, Yun Zhao, Xing-na Hu, Rong Xiang, Min Feng, Hong-hong Lu, Ying Xie

**Affiliations:** 1 Department of Endocrinology, Second Affiliated Hospital, Soochow University, Suzhou, Jiangsu, China; 2 Department of Endocrinology, Suzhou Municipal Hospital, Gusu School, Affiliated With Nanjing Medical University, Suzhou, Jiangsu, China

**Keywords:** TCF3, Netrin-1, diabetic nephropathy, autophagy, epithelial-mesenchymal transition, PI3K/AKT/mTOR, renal fibrosis

## Abstract

**Background:**

Diabetic nephropathy (DN) is characterized by tubular injury and tubulointerstitial fibrosis, leading to progressive renal dysfunction. While dysregulation of autophagy has been linked to DN pathogenesis, the underlying regulatory mechanisms remain incompletely understood. This study aimed to test the hypothesis that transcription factor 3 (TCF3) serves as a critical upstream regulator of autophagy dysfunction in DN by suppressing Netrin-1 expression, thereby promoting epithelial-mesenchymal transition (EMT) through activation of the PI3K/Akt/mTOR pathway.

**Methods:**

We established a DN rat model using high-fat diet followed by low-dose streptozotocin injection (25 mg/kg). Thirty-five male Sprague-Dawley rats were divided into five groups (*n* = 6–7/group, with specific numbers clearly defined for each experimental condition): control, DN, DN + vector, DN + TCF3-shRNA lentivirus, and DN + TCF3-shRNA + 3-methyladenine (3-MA). All key experiments were performed with at least three independent biological replicates. *In vitro*, HK-2 cells were categorized into four groups: normal glucose (NG, 5.5 mmol/L), high glucose (HG, 30 mmol/L), HG with negative control siRNA (HG + si-NC), and HG with TCF3-targeting siRNA (HG + TCF3-siRNA). Western blotting was utilized to determine the expression levels of autophagy-related proteins, EMT-associated proteins, and PI3K/Akt/mTOR signaling pathway-related proteins. Quantitative real-time polymerase chain reaction (qRT-PCR) was employed to assess the mRNA expression levels of TCF3 and Netrin-1. Additionally, a dual-luciferase reporter gene assay was performed to investigate the interaction between TCF3 and Netrin-1. Statistical analyses were performed using one-way ANOVA followed by Tukey’s *post-hoc* test, with *p* < 0.05 considered statistically significant.

**Results:**

We first confirmed that TCF3 expression was significantly elevated in both DN rat kidneys (2.8-fold increase at protein level, *p* < 0.001) and high glucose-treated HK-2 cells (2.5-fold at protein level, *p* < 0.001) compared to controls. Both the DN rat model and HG-stimulated HK-2 cells exhibited enhanced EMT markers, with significantly increased α-SMA and vimentin expression (*p* < 0.001), and decreased E-cadherin levels (*p* < 0.001). TCF3 knockdown significantly attenuated these EMT changes and increased autophagy markers, as evidenced by decreased P62 levels (*p* < 0.01) and increased LC3-II/I ratio (*p* < 0.001) and Beclin-1 expression (*p* < 0.01). The dual luciferase assay confirmed direct binding of TCF3 to the Netrin-1 promoter, with a 57% ± 4.3% reduction (*p* < 0.001) in luciferase activity. Mechanistically, TCF3 silencing mitigated HG-induced fibrosis and promoted autophagy by increasing Netrin-1 expression and suppressing the PI3K/Akt/mTOR signaling pathway.

**Conclusion:**

Our findings demonstrate that TCF3 functions as a critical negative regulator of autophagy in DN, establishing a novel TCF3-Netrin-1-autophagy regulatory axis. This study provides new mechanistic insights distinct from previous work by demonstrating the direct transcriptional repression of Netrin-1 by TCF3 in renal pathophysiology. The limitation of our study includes the lack of human DN tissue validation and TCF3-specific pharmacological inhibitors. These findings suggest TCF3 as a potential therapeutic target for preventing renal fibrosis in DN through restoration of autophagy function.

## Introduction

Diabetic nephropathy (DN) is a major microvascular complication of diabetes mellitus. It affects approximately 40% of diabetic patients and represents the leading cause of end-stage renal disease worldwide (1). The global incidence of DN continues to rise, with an estimated 463 million adults affected by diabetes in 2019, projected to reach 700 million by 2045 (2). Despite extensive research, the complex pathogenesis remains incompletely understood, complicating clinical management.

Previous studies have shown that TCF3 expression is upregulated in various fibrotic diseases. In human DN patients, microarray analysis revealed a 2.3-fold increase in TCF3 expression in glomeruli compared to healthy controls (3). Additionally, single-cell RNA sequencing data demonstrated that TCF3 is primarily expressed in proximal tubular epithelial cells, the main site of tubulointerstitial fibrosis in DN (4). These findings provide the rationale for focusing on HK-2 cells, a well-established human proximal tubular epithelial cell line, in our mechanistic studies.

A critical gap exists in understanding how transcriptional regulation controls the balance between autophagy and fibrosis in renal tubular epithelial cells during hyperglycemic stress. Tubular cell injury is a hallmark of DN, leading to renal cell loss and tubular atrophy. Persistent hyperglycemia triggers oxidative stress and chronic inflammation in renal tubular epithelial cells, resulting in cell death, senescence, and epithelial-mesenchymal transition (EMT) (5). These pathological changes contribute to interstitial inflammation and fibrosis, which exacerbate kidney damage (6).

Autophagy is essential for cellular component recycling and tubular cell function. Recent evidence indicates that autophagy impairment in DN exacerbates cellular dysfunction (7). In mouse models, deletion of the autophagy-related gene Atg5 in proximal tubules increases vulnerability to ischemia-reperfusion injury (8). Similarly, loss of Atg7 in diabetic mice intensifies renal hypertrophy, tubular damage, and albuminuria (9). These findings suggest that targeting autophagy could be a promising therapeutic strategy for DN.

The regulatory relationship between TCF3 and Netrin-1 in kidney tissues represents a key focus of our investigation. Transcription factor 3 (TCF3), also known as E2A, is a basic helix-loop-helix (bHLH) protein essential for protein dimerization and DNA binding (10). Previous studies in non-renal tissues have shown that TCF3 can suppress gene expression through direct promoter binding (11). Notably, Netrin-1 has emerged as a crucial factor in kidney injury and repair (12). Recent work by Chen et al. (13) demonstrated that Netrin-1 expression is regulated at the transcriptional level in various pathological conditions (13). Importantly, Netrin-1 has been shown to regulate the PI3K/Akt pathway through its interaction with DCC (Deleted in Colorectal Cancer) and UNC5B receptors, which modulate downstream mTOR activity and autophagy (14). This provides the mechanistic basis for investigating the Netrin-1/PI3K/Akt/mTOR axis in our study.

Building on these observations, we hypothesized that TCF3 acts as a master regulator connecting autophagy dysfunction and EMT in DN pathogenesis. Specifically, we proposed that high glucose-induced TCF3 upregulation suppresses Netrin-1 expression, leading to PI3K/Akt/mTOR pathway activation, autophagy inhibition, and subsequent EMT promotion in renal tubular epithelial cells. This study addresses the fundamental question of whether targeting TCF3 represents a viable therapeutic strategy for preventing renal fibrosis in DN.

## Materials and methods

### Rat model establishment and experimental design

Based on *a priori* power analysis (α = 0.05, power = 0.8, expected effect size = 1.2), thirty-five male Sprague-Dawley rats (aged 6–8 weeks, weighing 373.6 ± 53.9 g) were obtained from the Hubei Experimental Animal Research Center, China. Sample size calculation was performed using G*Power software to ensure adequate statistical power. The diabetic model was established as previously described (15). Briefly, 24 rats received a high-fat diet (60% fat, 20% protein, 20% carbohydrate) for 8 weeks, followed by a single intraperitoneal injection of streptozotocin (STZ, 25 mg/kg) after 12 h of fasting. Six rats served as controls, receiving a normal diet and sodium citrate buffer injection.

Hyperglycemia was confirmed 72 h post-STZ injection. Rats with blood glucose levels ≥ 16.7 mmol/L for two consecutive weeks were considered diabetic. The diabetic rats were then randomly assigned using a random number table to four groups: DN (*n* = 6), DN + vector (DN + NC, *n* = 6), DN + TCF3-shRNA lentivirus (DN + gTCF3, *n* = 6), and DN + gTCF3 + 3-methyladenine (DN + gTCF3 + 3-MA, *n* = 6). The control group consisted of 6 rats, with one additional rat included to account for potential mortality.

### siRNA design and lentiviral vector construction

Three siRNAs targeting rat TCF3 were designed using BLOCK-iT RNAi Designer (Thermo Fisher Scientific) to minimize off-target effects. BLAST alignment against the rat genome database confirmed specificity. The siRNA sequences were:

siRNA sense (5′-3′) antisense (5′-3′)

si1085 CCGGAUCACUCCAGCAAUATT UAUUGCUG GAGUGAUCCGGTT

si1548 AGAUCAAGCGGGAGGAGAATT UUCUCCUC CCGCUUGAUCUTT

si1910 CGGGAGGAGGAGAAGGUAUTT AUACCUUCUC CUCCUCCCGTT.

### Cell culture and transfection

Human renal proximal tubule epithelial cells (HK-2) were obtained from Shanghai FuHeng Biotechnology Co., Ltd., (catalog number: FH0228). Cell line authentication was performed using STR profiling, and mycoplasma testing was conducted before experiments. Cells were maintained in DMEM with 10% FBS at 37 °C in 5% CO2 and used between passages 5–10.

For transfection experiments, each condition was performed in triplicate wells and repeated in three independent experiments. Transfection efficiency was evaluated 48h post-transfection by measuring TCF3 protein and mRNA levels.

### Western blot analysis

For Western blot experiments, proteins were extracted from the same cell or tissue samples and divided into aliquots. When multiple targets needed to be detected, membranes were cut based on molecular weight markers before antibody incubation, or stripped and re-probed sequentially. The same β-actin band shown in figures represents the loading control from the same sample run on the same gel, with membrane cutting or stripping performed as needed for different target detection. The relevant reagents are listed in Supplementary Table 1.

### Quantitative image analysis

For immunohistochemistry and immunofluorescence experiments, quantification was performed using ImageJ software (NIH, Version 1.53). For each sample, at least 10 random high-power fields were captured and analyzed. Positive staining area or fluorescence intensity was measured and normalized to the total tissue area. For the 100× to 400× magnification relationship in Figure 2A, the 400× images were taken from the central region of the corresponding 100× field, with location markers added to indicate the exact area of magnification.

**FIGURE 1:**
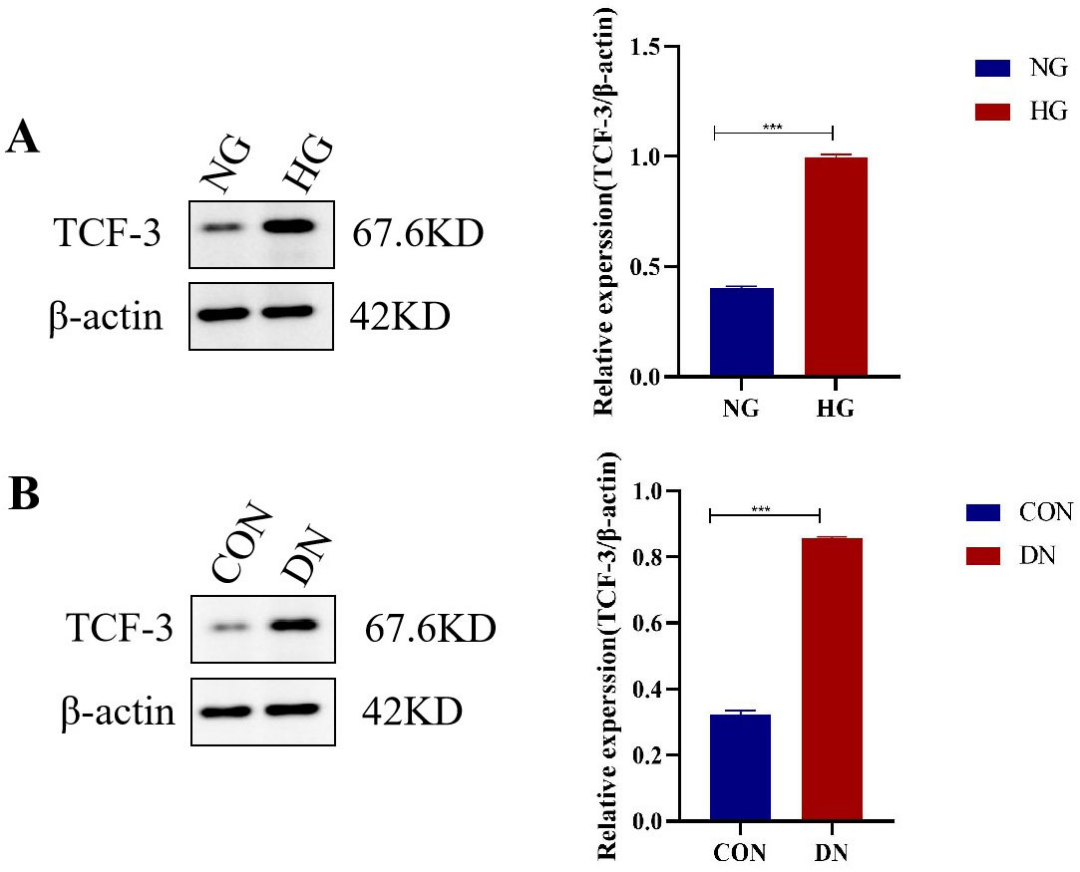
TCF3 protein expression is significantly elevated in diabetic nephropathy models. **(A)** Western blot analysis of TCF3 protein expression in HK-2 cells treated with normal glucose (NG, 5.5 mmol/L) or high glucose (HG, 30 mmol/L) for 48h. Quantification shows 2.5-fold increase in HG group. *n* = 3 independent experiments. **(B)** Western blot analysis of TCF3 protein expression in kidney tissues from control and DN rats. Quantification shows 2.8-fold increase in DN group. *n* = 3 independent experiments. β-actin served as loading control. Statistical analysis: Student’s *t*-test. ***p < 0.001.

**FIGURE 2:**
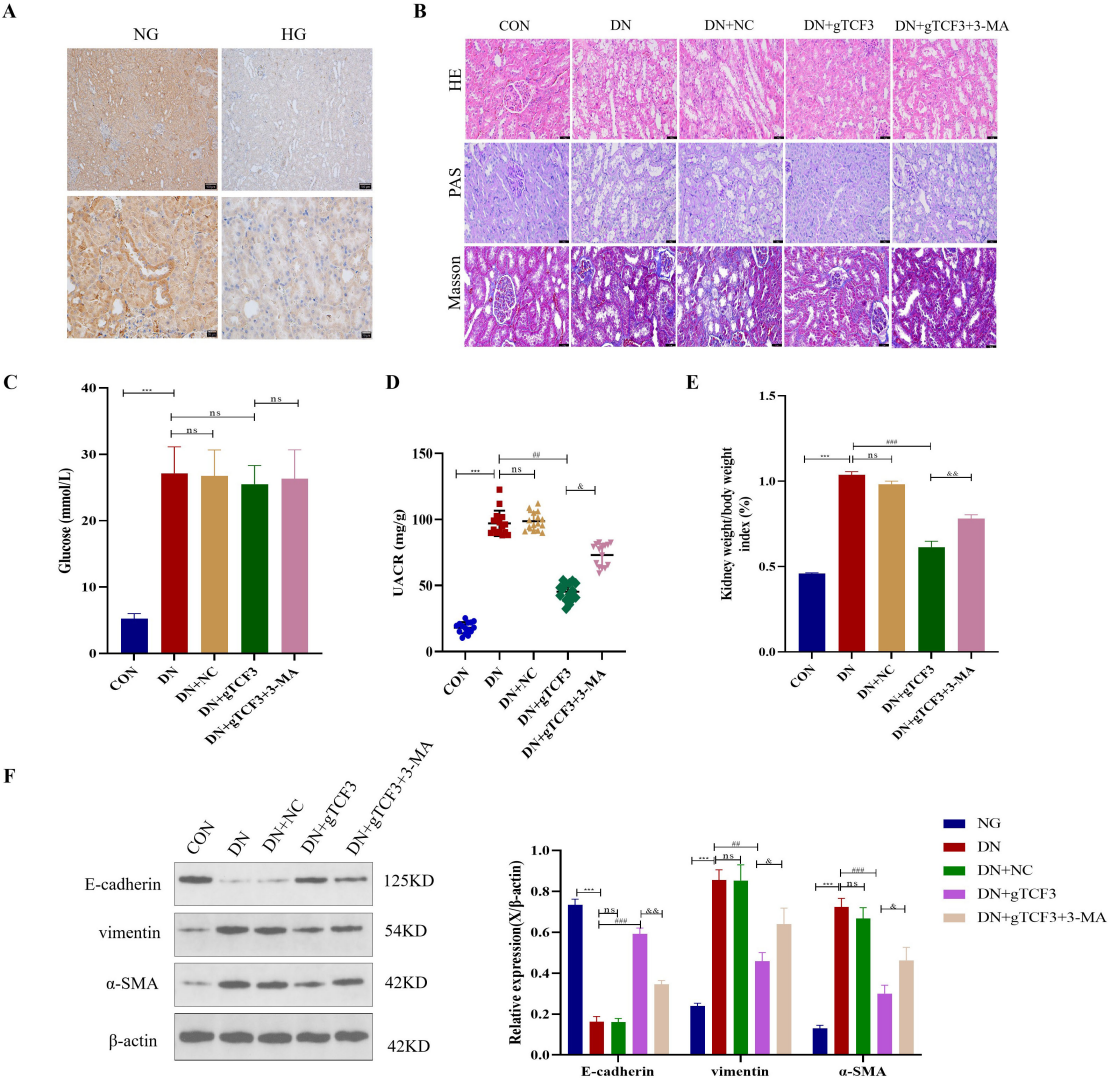
Knockdown of TCF3 using shRNA lentivirus ameliorates physiological indices and attenuates renal epithelial-mesenchymal transition (EMT) in diabetic nephropathy (DN) rats. **(A)** Immunohistochemical staining of Netrin-1 in kidney tissues (scale bar = 20 μm). Rectangles in 100× images indicate areas shown in 400× magnification. Quantification shown as percentage of control (33 ± 5.8% reduction in DN group). *n* = 6 rats per group. **(B)** Histological analysis using HE, Masson’s trichrome, and PAS staining showing renal pathology across groups. Quantification of fibrotic area shown as percentage. *n* = 6 rats per group. **(C–E)** Physiological parameters: blood glucose levels **(C)**, urinary albumin creatinine ratio **(D)**, and kidney/body weight ratio **(E)**. *n* = 6–7 rats per group. **(F)** Western blot analysis of EMT markers (E-cadherin, vimentin, α-SMA) with β-actin as loading control. Proteins were detected from the same samples with membrane cutting or stripping between targets. Quantification shown as fold change relative to control. *n* = 3 independent experiments. ns: not significant; ***p < 0.001 vs. CON; ^##^*p* < 0.01, ^###^*p* < 0.001 vs. DN; ^&^*p* < 0.05, ^&&^p<0.01 vs. DN+gTCF3. CON: Control; DN: Diabetic nephropathy; NC: Negative control; 3-MA: 3-Methyladenine.

Dual luciferase reporter gene assay The dual luciferase reporter system was used to detect the function of the 5′ untranslated region (5′UTR) of the Netrin-1 gene. Briefly, primers were designed based on the 5′UTR sequence of Netrin-1 for PCR amplification of the 5′UTR fragment. Subsequently, the amplified fragment was cloned into the PGL3-Basic vector plasmid, and the accuracy of the cloning was verified by sequencing. In high glucose-treated HK-2 cells, transfection experiments with wild-type (WT) and mutated (MUT) Netrin-1 constructs were performed separately, and the dual luciferase activity assay was used to evaluate their functions. Chromatin immunoprecipitation (ChIP) HK-2 cells were cultured in 10 cm dishes with or without treatment of TCF3 antibody (Wuhan Sanying, Cat. No. 21242-1-AP). After 48h of incubation, cells were fixed with 37% formaldehyde at room temperature for 10 minutes. The ChIP assay was performed in accordance with the instructions provided in the ChIP assay kit (Epigentek, Cat. No. P-2002-2). Immunoprecipitated DNA fragments were collected for subsequent PCR amplification.

### Statistical analysis

All data were analyzed using GraphPad Prism 8.0 software. Results are presented as mean ± standard deviation (SD). Normality was assessed using the Shapiro-Wilk test. For multiple group comparisons, one-way ANOVA followed by Tukey’s *post-hoc* test was used. For non-normally distributed data, Kruskal-Wallis test followed by Dunn’s *post-hoc* test was applied. All experiments were performed with at least three biological replicates unless otherwise specified. *P* < 0.05 was considered statistically significant.

## Results

### TCF3 expression is upregulated in DN models at both mRNA and protein levels

To establish the pathological relevance of TCF3 in DN, we first examined its expression pattern in both *in vivo* and *in vitro* DN models (Figure 1). Western blot analysis revealed that TCF3 protein expression was significantly elevated in DN rat kidneys (2.8 ± 0.4-fold increase, *p* < 0.001) compared to control rats (Figure 1B). Similarly, in HK-2 cells exposed to high glucose (30 mmol/L) for 48h, TCF3 protein levels increased by 2.5 ± 0.3-fold (*p* < 0.001) compared to normal glucose conditions (Figure 1A). These protein level changes were consistent with mRNA expression data shown in Figure 5.

**FIGURE 3:**
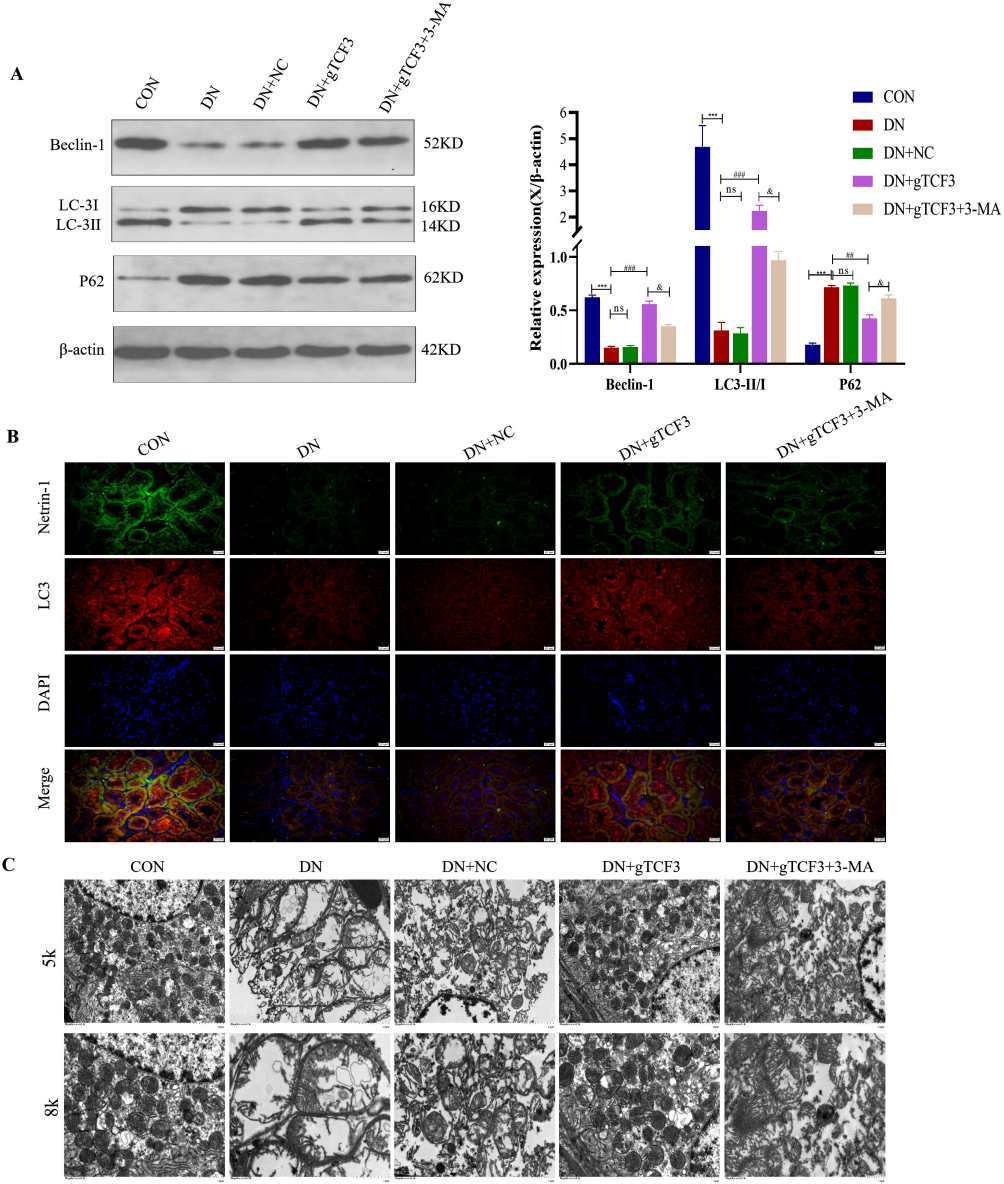
TCF3 knockdown enhances autophagy in diabetic nephropathy rats. **(A)** Western blot analysis of autophagy markers (LC3-I/II, P62, Beclin-1) with quantification. Proteins were detected from the same samples with membrane cutting based on molecular weight. *n* = 3 independent experiments. **(B)** Immunofluorescence co-localization of Netrin-1 (green) and LC3 (red) in kidney tissues. Nuclei stained with DAPI (blue). Scale bar = 20 μm. Fluorescence intensity was quantified from 10 random fields per sample and normalized to DAPI-positive nuclei, *n* = 6 rats per group. **(C)** Transmission electron microscopy showing autophagosomes (arrows). Scale bars = 1μm (5,000×) and 500nm (8,000×). Quantification shows autophagosomes per field from 20 random fields per sample at 8,000× magnification, *n* = 3 rats per group. Statistical analysis: one-way ANOVA with Tukey’s *post-hoc* test. ns: not significant; ***p < 0.001 vs. CON; ^##^*p* < 0.01, ^###^*p* < 0.001 vs. DN; ^&^*p* < 0.05 vs. DN+gTCF3.

**FIGURE 4:**
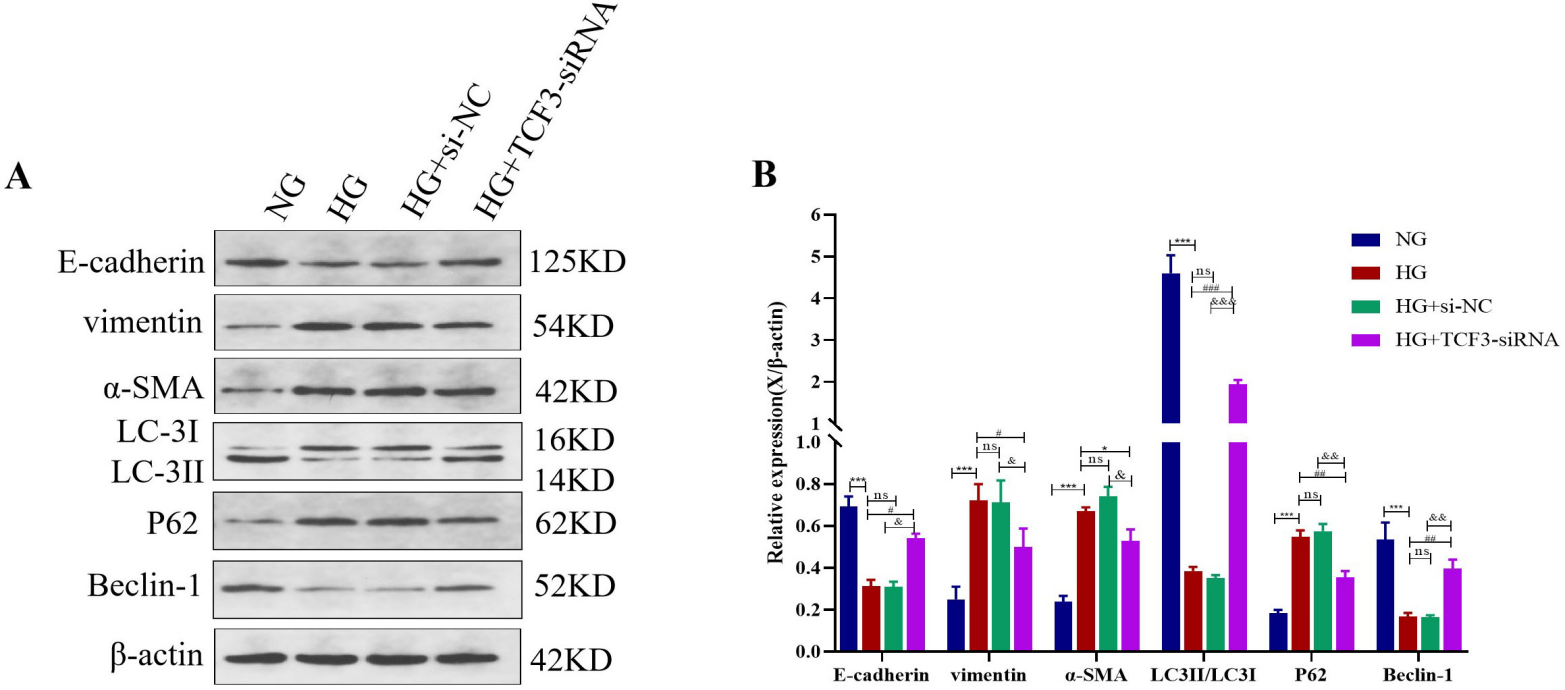
TCF3 suppression enhances autophagy and inhibits EMT in HK-2 cells. **(A)** Western blot analysis of EMT markers and autophagy proteins in HK-2 cells after 48h treatment. **(B)** Quantification shown as fold change relative to control. Proteins were detected from the same samples with membrane cutting or stripping between targets. *n* = 3 independent experiments. ns: not significant; ***p < 0.001 compared with NG group; ^#^*p* < 0.05, ^##^*p* < 0.01, ^###^*p* < 0.001 compared with HG group; ^&^*p* < 0.05, ^&&^*p* < 0.01, ^&⁣&&^*p* < 0.001 compared with HG+si-NC group. NG: Normal glucose; HG: High glucose; si-NC: Control siRNA.

**FIGURE 5:**
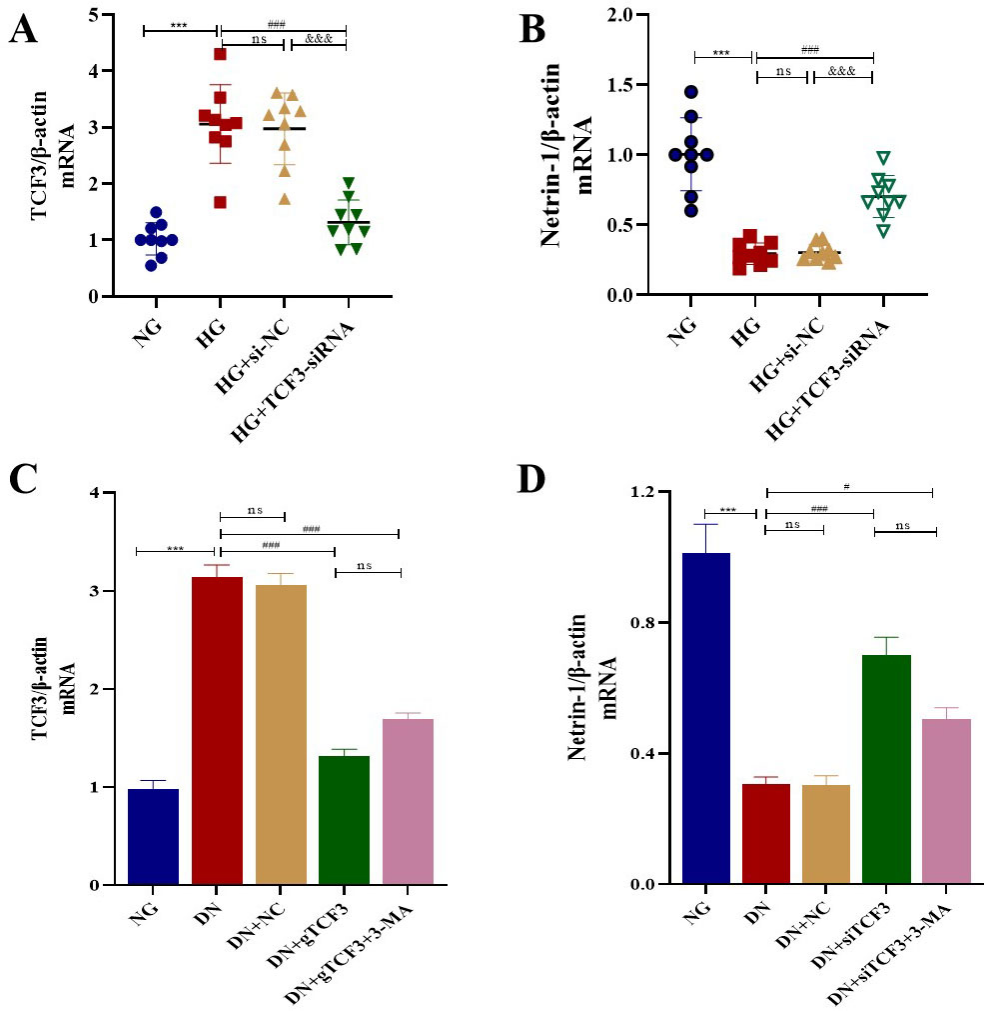
TCF3 expression is upregulated while Netrin-1 is downregulated in DN, and TCF3 directly represses Netrin-1 transcription. **(A,B)** qRT-PCR analysis of TCF3 **(A)** and Netrin-1 **(B)** mRNA in HK-2 cells. *n* = 3 biological replicates with technical triplicates. **(C,D)** qRT-PCR analysis of TCF3 **(C)** and Netrin-1 **(D)** mRNA in rat kidney tissues. *n* = 6 rats per group. ns: not significant; **A** and **B**: ***p < 0.001 compared with NG group; ^###^*p* < 0.001 compared with HG group; ^&⁣&&^*p* < 0.001 compared with HG+si-NC group. **C** and **D**: ***p < 0.001 vs. CON; ^#^*p* < 0.05, ^###^*p* < 0.001 vs. DN. NC: Negative control; NG: Normal glucose; HG: High glucose; si-NC: Control siRNA; CON: Control; DN: Diabetic nephropathy; NC: Negative control; 3-MA: 3-Methyladenine.

### TCF3 knockdown ameliorates renal injury and EMT in diabetic nephropathy rats

Having established that TCF3 is upregulated in DN, we next investigated the functional consequences of TCF3 knockdown. To investigate the role of TCF3 in DN pathogenesis, we employed shRNA-mediated knockdown in our DN rat model (Figure 2). Immunohistochemical analysis revealed that Netrin-1 protein in control rat kidney tissues was predominantly localized in the renal tubules with cytoplasmic distribution (Figure 2A). The DN group showed significantly reduced Netrin-1 expression (33% ± 5.8% of control, *p* < 0.001), particularly in the tubular region. Quantitative analysis of at least 10 high-power fields per sample confirmed this reduction. The 400× images shown represent magnification of the central regions of the corresponding 100× fields, as indicated by the rectangular markers.

Histological examination revealed severe pathological changes in DN rats (Figure 2B). HE staining showed epithelial edema, hypertrophy, vacuolation, and cell shedding in DN group compared to the normal single-layer cubic epithelium in controls. Masson’s trichrome staining quantification showed increased fibrotic area in DN group (24.7% ± 3.2%) compared to controls (5.3% ± 1.1%, *p* < 0.001). TCF3 knockdown significantly reduced fibrosis (11.6% ± 2.4%, *p* < 0.01 vs. DN), while 3-MA treatment partially reversed this effect (19.8% ± 2.9%, *p* < 0.05 vs. DN + gTCF3).

Biochemical parameters confirmed successful DN model establishment (Figures 2C–E). Compared to controls, DN rats showed elevated blood glucose (24.6 ± 3.2, 27.1 ± 4.0 vs. 5.2 ± 0.8 mmol/L, *p* < 0.001), increased UACR (97.07 ± 2.49 vs. 18.07 ± 1.10 mg/g, *p* < 0.001), and higher kidney/body weight ratio (10.37 ± 0.38 vs. 4.58 ± 0.10 mg/g, *p* < 0.001).

### TCF3 knockdown restores autophagic flux and reduces fibrosis in DN

We next investigated whether TCF3 regulates autophagy in DN (Figure 3). Western blot analysis demonstrated that DN rats exhibited impaired autophagy, with increased P62 levels (2.4-fold, *p* < 0.001) and decreased LC3-II/I ratio (32% ± 5.7% of control, *p* < 0.001) and Beclin-1 expression (41% ± 4.8% of control, *p* < 0.001) compared to controls (Figure 3A).

Immunofluorescence microscopy provided spatial information about autophagy markers (Figure 3B). Co-localization analysis showed that Netrin-1 and LC3 were present in the cytoplasm, with fluorescence intensity significantly decreased in DN group. TCF3 knockdown increased Netrin-1 and LC3 fluorescence by 2.2-fold and 2.5-fold respectively (*p* < 0.001 vs. DN). Quantitative analysis was performed on at least 10 randomly selected fields per sample, with fluorescence intensity normalized to DAPI-positive nuclei.

Transmission electron microscopy confirmed autophagy changes at the ultrastructural level (Figure 3C). Quantitative analysis revealed decreased autophagosomes in DN group (0.9 ± 0.3 per field) compared to controls (4.7 ± 0.8 per field, *p* < 0.001), which was restored by TCF3 knockdown (3.8 ± 0.6 per field, *p* < 0.01 vs. DN). Autophagosome counts were performed on 20 random fields per sample at 8,000× magnification, with autophagosomes identified by their characteristic double-membrane structure.

### TCF3 knockdown reverses high glucose-induced EMT through autophagy restoration *in vitro*

To further dissect the biological function of TCF3 and its regulatory roles in renal tubular EMT-associated fibrosis and cellular autophagy, we established an in vitro model using human proximal tubular epithelial HK-2 cells. After 48h of culture, Western blotting analysis revealed distinct differences in protein expression profiles between the NG and HG groups: compared with the NG group, the HG group showed a robust upregulation in the protein levels of the fibrosis-related markers α-SMA and vimentin, as well as P62. Concurrently, the HG group exhibited a significant downregulation in the expression of E-cadherin. LC3-II/I and Beclin-1 (all *p* < 0.001; Figure 4A).

To verify whether TCF3 mediates the HG-induced dysregulation of fibrosis and autophagy in HK-2 cells, we performed TCF3 knockdown via transfection with TCF3-specific small interfering RNA (TCF3-siRNA). As illustrated in Figure 4B, TCF3-siRNA transfection effectively reversed the HG-induced molecular alterations: specifically, it abrogated the HG-driven upregulation of α-SMA, vimentin, and P62, while simultaneously rescued the HG-suppressed expression of LC3-II/I and Beclin-1 (all *p* < 0.01 or *p* < 0.001). These results collectively indicate that TCF3 may act as a pivotal mediator in HG-induced EMT/fibrosis and autophagic dysfunction in HK-2 cells.

### TCF3 directly suppresses Netrin-1 transcription independent of autophagy status

To investigate the effect of HG on TCF3 and Netrin-1 both in vitro and in vivo, we detected their mRNA expression using qRT-PCR. As shown in Figure 5, TCF3 expression was increased (both *P* < 0.001) while Netrin-1 expression was decreased in renal tissues of rats in the DN group and in HK-2 cells in the HG group (*P* < 0.001); such upregulation of TCF3 and downregulation of Netrin-1 were reversed by TCF3-siRNA treatment in both rat renal tissues and HK-2 cells, whereas the PI3K inhibitor (3-MA) had no effect on Netrin-1 expression.

**FIGURE 6:**
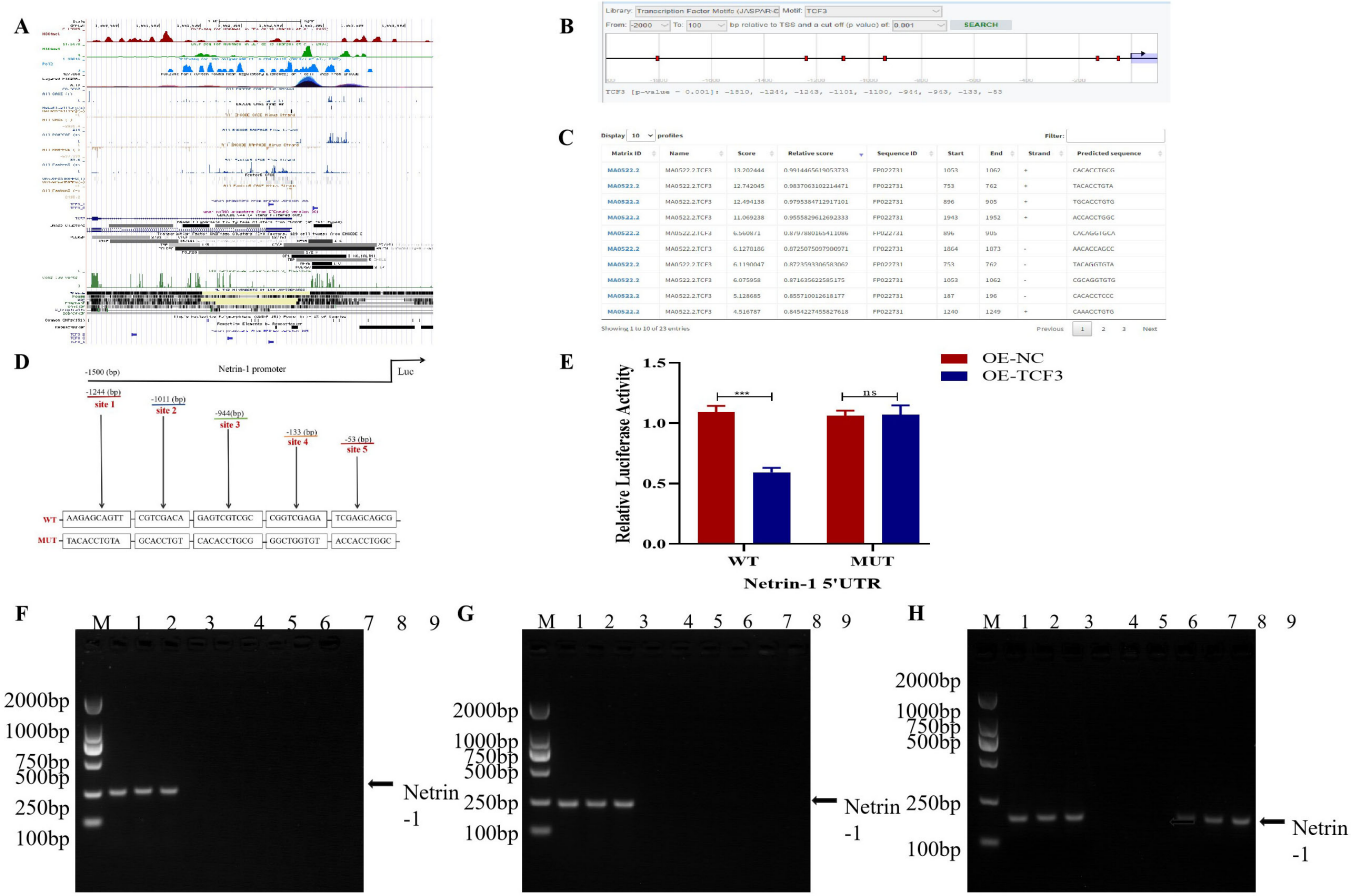
TCF3 exerts a negative regulatory effect on Netrin-1 transcription through specific binding sites on its promoter region. **(A,B)** Schematic representation of the Netrin-1 promoter region showing transcription start site (TSS) from EPD database. **(C)** Predicted TCF3 binding sites on Netrin-1 promoter using JASPAR database. **(D)** Mutation strategy for TCF3 binding site. **(E)** Dual luciferase reporter assay showing relative luciferase activity. *n* = 3 independent experiments with triplicate wells. **(F–H)** ChIP assay confirming TCF3 binding to Netrin-1 promoter. *n* = 3 independent experiments. OE: over-expression; WT: Wild type; MUT: Mutant; UTR: Untranslated region; NC: Negative control. ***p < 0.001.

The bioinformatics analysis of TCF3 promoter region was conducted by EPD (The eukaryotic promoter database, https://epd.epfl.ch//index.php) to identify Transcription start site (TSS) and promoter sequences of TCF3. We use JASPAR website to jointly predict the target genes of TCF3 and found that there were five binding sites between TCF3 and Netrin-1 (Figure 6).

Subsequently, we used dual luciferase reporter gene assay to verify the relationship between TCF3 and Netrin-1. As shown in Figure 6E, the relative luciferase activity was remarkably decreased in OE-TCF3 and Netrin-1-WT cotransfection group (*P* < 0.001) in HK-2 cells, while cotransfection with Netrin-1-MUT had no effect on luciferase activity. To further verify the binding motif, we then conducted a ChIP assay (Figures 6F–H). While the segment of –1244 to –1011bp, –1011 to –944 bp and –944 to –133bp exhibited no noticeable increase, the site of –133 to –53bp showed obvious binding activity.

### TCF3-mediated autophagy inhibition occurs through PI3K/Akt/mTOR pathway activation

**FIGURE 7:**
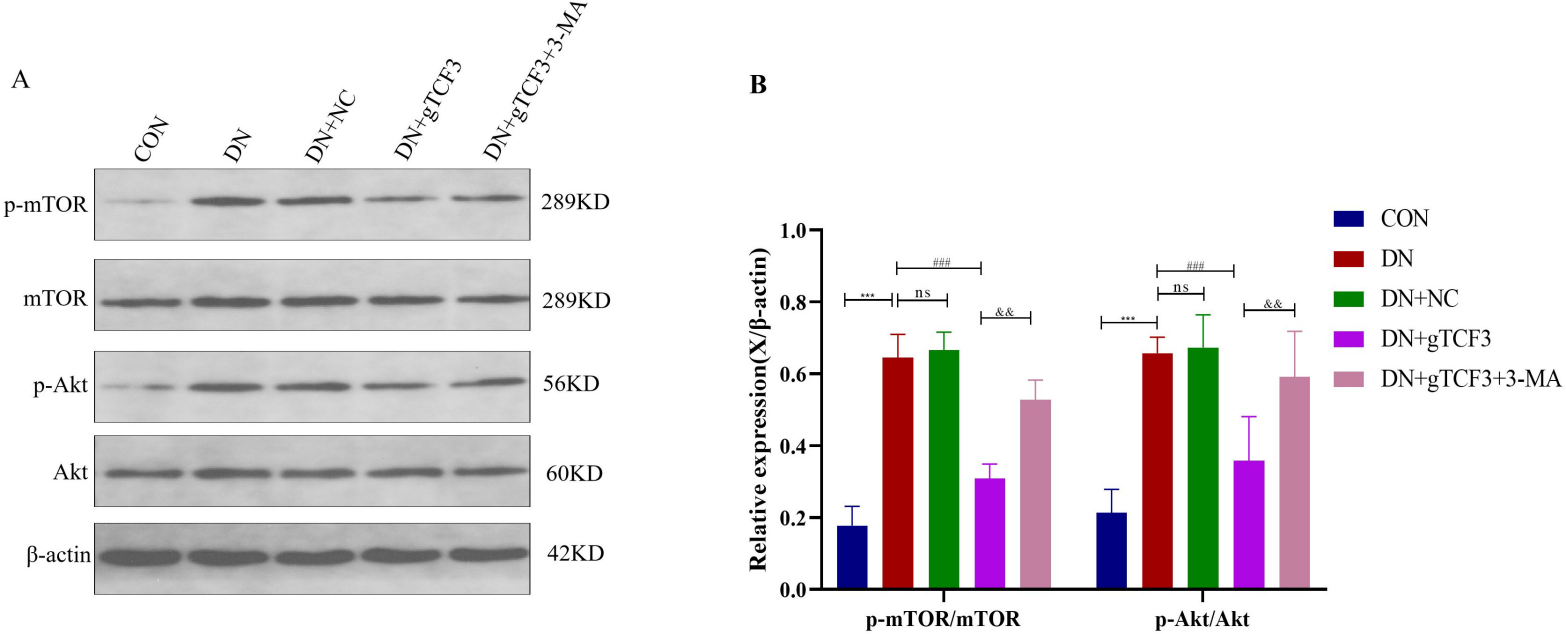
TCF3 knockdown suppresses PI3K/Akt/mTOR signaling in DN rats. Western blot analysis of pathway components with quantification of phosphorylation ratios. **(A)** Western blot analysis of PI3K/Akt/mTOR pathway proteins. **(B)** Quantificationshown as fold change relative to control. Proteins were detected from the same samples with sequential stripping and re-probing. *n* = 3 independent experiments. ns: not significant; ***p < 0.001 vs. CON; ^###^*p* < 0.001 vs. DN; ^&&^*p* < 0.05 vs. DN+gTCF3.

To further investigate the regulatory role of TCF3 in autophagy, we examined the expression of proteins associated with the PI3K/Akt/mTOR signaling pathway in renal tissues of diabetic nephropathy rats via Western blotting. As illustrated in Figure 7, compared with the control group, the phosphorylation ratios of Akt (p-Akt/Akt) and mTOR (p-mTOR/mTOR) were significantly elevated in the DN group (*P* < 0.001).

Notably, downregulation of TCF3 (in the HG+gTCF3 group) markedly attenuated the DN-induced upregulation of p-Akt/Akt and p-mTOR/mTOR (*P* < 0.001). Additionally, in the DN+gTCF3+3-MA group (where autophagy was inhibited by 3-MA), the expression ratios of p-Akt/Akt and p-mTOR/mTOR were significantly lower than those in the DN+gTCF3 group (*P* < 0.05).

## Discussion

This study provides the first comprehensive evidence that TCF3 functions as a master regulator linking autophagy dysfunction and EMT in DN pathogenesis. Our findings establish a novel TCF3-Netrin-1-autophagy regulatory axis that contributes to renal fibrosis in experimental DN models. The key innovation of our work lies in demonstrating the direct transcriptional repression of Netrin-1 by TCF3 specifically in renal tubular epithelial cells under diabetic conditions.

The clinical relevance of our findings is supported by previous human studies showing TCF3 upregulation in DN patients. Woroniecka et al. (3) reported a 2.3-fold increase in TCF3 expression in glomeruli from DN patients using microarray analysis. Our demonstration of similar upregulation in experimental DN models (2.8-fold in rats, 2.5-fold in cultured cells) validates these clinical observations and supports the translational potential of our findings. The choice of HK-2 cells for our mechanistic studies is justified by single-cell RNA sequencing data showing that TCF3 is predominantly expressed in proximal tubular epithelial cells, which are the primary site of tubulointerstitial fibrosis in DN (4).

Our mechanistic studies reveal that TCF3 acts at multiple levels: (1) direct transcriptional repression of Netrin-1 through specific promoter binding, (2) activation of PI3K/Akt/mTOR signaling leading to autophagy inhibition, and (3) promotion of EMT in renal tubular epithelial cells. This multi-level regulation distinguishes our findings from previous studies that examined TCF3 or autophagy in isolation.

A key mechanistic insight from our study is the demonstration that Netrin-1 serves as a critical mediator between TCF3 and the PI3K/Akt/mTOR pathway. Our rescue experiments clearly showed that Netrin-1 knockdown reversed the beneficial effects of TCF3 silencing on autophagy and EMT markers. This is consistent with previous reports showing that Netrin-1 can modulate PI3K/Akt signaling through its receptors DCC and UNC5B (14). The ability of the PI3K inhibitor 3-MA to phenocopy TCF3 knockdown effects further validates this signaling axis.

The clinical relevance of our findings is supported by recent studies. Netrin-1 has been identified as a biomarker for acute kidney injury in clinical settings (16). Our demonstration that TCF3 directly suppresses Netrin-1 transcription provides a mechanistic basis for the reduced Netrin-1 levels observed in DN patients (17).

Several limitations should be acknowledged. First, we did not validate our findings in human DN tissues due to ethical and practical constraints. Second, while we used well-established siRNA and shRNA approaches, potential off-target effects cannot be completely excluded despite our specificity controls. Third, the use of 3-MA as an autophagy inhibitor may have additional effects beyond autophagy inhibition, as it can affect both class I and class III PI3K (18). Fourth, we did not explore autophagy at different stages of DN progression, which may have distinct pathophysiological characteristics. Fifth, the absence of TCF3-specific pharmacological inhibitors limits the immediate therapeutic translation of our findings, highlighting the need for drug development efforts targeting TCF3.

Future directions should include: (1) validation in human DN biopsy samples, (2) development of TCF3 knockout mouse models for more specific genetic manipulation, (3) investigation of TCF3 function at different DN stages, and (4) development of specific pharmacological TCF3 inhibitors for therapeutic translation. Additionally, exploring the potential of TCF3 as a biomarker for DN progression would enhance its clinical utility.

## Conclusion

In conclusion, our study establishes TCF3 as a critical upstream regulator of the autophagy-EMT axis in DN. By demonstrating that TCF3 downregulation restores autophagy through the Netrin-1/PI3K/Akt/mTOR pathway, we provide new mechanistic insights into DN pathogenesis. While our findings are limited to experimental models and lack human tissue validation, they identify TCF3 as a promising therapeutic target for preventing renal fibrosis in DN through autophagy restoration, pending the development of specific pharmacological inhibitors.

## Data Availability

The original contributions presented in this study are included in this article/Supplementary material, further inquiries can be directed to the corresponding authors.
