# Supplementary material for: TCF3 downregulation alleviates renal fibrosis via PI3K/Akt/mTOR pathway inhibition and autophagy restoration in diabetic nephropathy
Source: Front Med (Lausanne). 2025 Dec 1;12:1547410. doi: 10.3389/fmed.2025.1547410 (PMC12702734; doi:10.3389/fmed.2025.1547410)
Supplement: Supplementary file 1 [file Table_1.docx]

## Supplementary Information

### Table S1. Antibodies used in this study

| **Antibody** | **Source** | **Catalog #** | **Dilution** | **Application** |
| --- | --- | --- | --- | --- |
| TCF3 | Proteintech | 21242-1-AP | 1:1000 (WB), 1:200 (IHC) | Western blot, Immunohistochemistry |
| Netrin-1 | Santa Cruz Biotechnology | sc-6535 | 1:500 (WB), 1:100 (IF) | Western blot, Immunofluorescence |
| LC3 | Proteintech | 14600-1-AP | 1:3000 (WB), 1:100 (IF) | Western blot, Immunofluorescence |
| P62/SQSTM1 | Affinity Biosciences | AF5384 | 1:1000 (WB) | Western blot |
| Beclin-1 | Affinity Biosciences | AF5128 | 1:1000 (WB) | Western blot |
| E-cadherin | Proteintech | 20874-1-AP | 1:2000 (WB) | Western blot |
| vimentin | Proteintech | 10366-1-AP | 1:5000 (WB) | Western blot |
| α-SMA | Proteintech | 80008-1-RR | 1:2000 (WB) | Western blot |
| mTOR | Proteintech | 66888-1-Ig | 1:5000 (WB) | Western blot |
| p-mTOR (Ser2448) | Affinity Biosciences | AF3308 | 1:1000 (WB) | Western blot |
| Akt | Cell Signaling Technology | 2938S | 1:1000 (WB) | Western blot |
| p-Akt (Ser473) | Affinity Biosciences | AF0016 | 1:1000 (WB) | Western blot |
| PI3K p85 | Cell Signaling Technology | 4257S | 1:1000 (WB) | Western blot |
| β-actin | Proteintech | 60008-1-Ig | 1:10000 (WB) | Western blot (loading control) |
| GAPDH | Proteintech | 60004-1-Ig | 1:10000 (WB) | Western blot (loading control) |
| Anti-rabbit IgG-HRP | Jackson ImmunoResearch | 111-035-003 | 1:5000 (WB) | Secondary antibody |
| Anti-mouse IgG-HRP | Jackson ImmunoResearch | 115-035-003 | 1:5000 (WB) | Secondary antibody |
| Anti-goat IgG-HRP | Jackson ImmunoResearch | 705-035-003 | 1:5000 (WB) | Secondary antibody |
| Alexa Fluor 488 anti-rabbit | Invitrogen | A-11008 | 1:500 (IF) | Secondary antibody |
| Alexa Fluor 594 anti-mouse | Invitrogen | A-11005 | 1:500 (IF) | Secondary antibody |

**Abbreviations:** WB: Western blot; IHC: Immunohistochemistry; IF: Immunofluorescence; HRP: Horseradish peroxidase

**Note:** All primary antibodies were diluted in 5% BSA in TBS-T (0.1% Tween-20) for Western blot or 3% BSA in PBS for immunofluorescence. Secondary antibodies were diluted in 5% non-fat milk in TBS-T for Western blot or 1% BSA in PBS for immunofluorescence.
